# Synthesis and Characterization of Bi_4_Ti_3_O_12_ Nanoparticles Obtained via Pulsed Laser Ablation in Liquids

**DOI:** 10.3390/ma16237451

**Published:** 2023-11-30

**Authors:** Linda Viviana García-Quiñonez, Dario Colorado-Garrido, Adolfo Collado-Hernández, Daniel Arturo Acuña-Leal, Guadalupe Alan Castillo-Rodríguez, Daniel Fernández-González, Eduardo Martínez-Guerra, María Isabel Mendívil-Palma, Cristian Gomez-Rodríguez

**Affiliations:** 1 Centro de Investigación en Recursos Energéticos y Sustentables (CIRES), Universidad Veracruzana, Avenue Universidad Veracruzana Km 7.5, Col. Santa Isabel I, Coatzacoalcos 96538, Mexico; lingarcia@uv.mx (L.V.G.-Q.); dcolorado@uv.mx (D.C.-G.); 2 Faculty of Physics and Mathematics, Universidad Autónoma de Nuevo León, San Nicolás de los Garza 66455, Mexico; adolfo.colladoh@uanl.edu.mx (A.C.-H.); daniel.lfupap@outlook.com (D.A.A.-L.); 3 Universidad Politécnica de Apodaca, Avenue Politécnica 2331, El Barretal, Apodaca 66600, Mexico; 4 Facultad de Ingeniería Mecánica y Eléctrica (FIME), Universidad Autónoma de Nuevo León (UANL), San Nicolás de los Garza 66450, Mexico; alan.castillo.rdz@gmail.com; 5 Nanomaterials and Nanotechnology Research Center (CINN-CSIC), Universidad de Oviedo (UO), Principado de Asturias (PA), Avda. de la Vega, 4-6, 33940 El Entrego, Spain; d.fernandez@cinn.es; 6 Centro de Investigación en Materiales Avanzados, S.C. (CIMAV) Subsede Monterrey, Alianza Norte 202, Parque PITT, Apodaca 66628, Mexico; eduardo.martinez@cimav.edu.mx; 7 Departamento de Mecánica, Facultad de Ingeniería, Campus Coatzacoalcos, Universidad Veracruzana, Avenue Universidad Km 7.5 Col. Santa Isabel I, Coatzacoalcos 96538, Mexico

**Keywords:** bismuth titanate, nanoparticles, pulsed laser ablation, Nd:YAG laser

## Abstract

Bismuth titanate (BTO) nanoparticles were obtained by pulsed laser ablation in liquid media (PLAL). Distilled water, ethanol, isopropanol, and acetone were used as media for laser ablation experiments, in which the colloidal solutions were obtained. Laser ablation was carried out using the second harmonic and fundamental wavelength of a pulsed Nd:YAG laser (532 nm and 1064 nm, respectively) with laser fluences of 25 and 12 mJ/cm^2^, respectively. Transmission electron microscopy was utilized for morphological characterization. BTO nanoparticles obtained have spherical shapes with orthorhombic structure and the average size distribution depended on the liquid media nature. In alcohols, BTO NPs were spherical with a carbon layer around them. X-ray diffraction, UV-Vis absorption spectra, and X-ray photoelectron spectroscopy were used to confirm the structural, optical, and elemental properties of the ablated products. The presented results show that PLAL is a viable technique for the synthesis of high-quality BTO nanoparticles with enhanced optical properties for possible applications in photocatalysis.

## 1. Introduction

Bismuth titanate compounds (BTOs), such as Bi_4_Ti_3_O_12_ and Bi_2_Ti_2_O_7_, are the simplest members of the Aurivillius family, which consist of (Bi_2_O_2_)^2+^ sheets alternating with (Bi_2_Ti_3_O_10_)^2−^ pseudo-perovskite layers [1,2]. Due to this particular structure, BTO compounds exhibit interesting physical properties such as ferroelectricity, high Curie temperature (675 °C) [2,3,4,5,6,7], and a band gap of 3.08 eV, depending on the morphology and synthesis route [8,9], among others. These properties enable a wide spectrum of technological applications, e.g., in catalysis [8,9,10,11,12,13,14,15]. BTOs have been synthesized as thin films [16,17,18] and nanostructured powders with solid-state and wet-chemistry synthesis routes, which include calcination [19], coprecipitation [20], hydrothermal [21], sonochemical [22], and mechanochemical methods [23,24], among others.

On the other hand, pulsed laser ablation in liquid (PLAL) is a novel method for the generation of particles with sizes ranging from the micro- to nanometer scale directly from bulk materials. This technique consists of a pulsed laser focused on a bulk material, which is immersed in a liquid medium. The mechanism of nanoparticle generation by PLAL starts with the interaction of the bulk material with a laser pulse that has a fluence greater than its ablation threshold [25,26], which forms a high-temperature plasma around the surface of the material [25,26,27,28,29,30]; rapid cooling produces a cavitation bubble, where the ablated products are contained and grow until the cavitation bubble collapses and the nanoparticles are released into the solution [31,32,33]. Also, pulsed laser ablation in liquids is an advantageous synthesis method, since nanoparticles are produced in a single step, and the method does not require ligands or surfactants, which is attractive for catalysis and analytical chemistry applications [25]. Some examples of nanoparticles produced by this method include ceramic oxides [34,35], and silicon-based [36] and metallic nanoparticles [37].

On the other hand, laser parameters, as well as the physicochemical properties of the target material and liquid medium, play an important role in the stages of nanoparticle generation [33,38]; for example, the fluence and pulse width of the laser, along with thermal conductivity of the target material, are the constituent properties of the plasma and the cavitation bubble [38]. Furthermore, the selection of the liquid medium has a direct impact on the properties of the nanoparticles, since laser irradiation can induce decomposition of the media, thus altering the size, surface chemistry, composition, and morphology of the ablated products; for instance, laser ablation in distilled water can produce oxidation and hydroxylation, whereas PLAL in organic solvents, such as acetone, ethanol and isopropanol, can induce carbonization of metallic nanoparticles, encapsulate nanoparticles with carbon, and limit the sizes of the ablated products [39].

In this work, BTO nanoparticles were synthesized using pulsed laser ablation in different liquid media (distilled water (DW), ethanol, isopropanol, and acetone) and their morphological, structural, elemental, and optical properties were studied.

## 2. Materials and Methods

**Figure 1:**
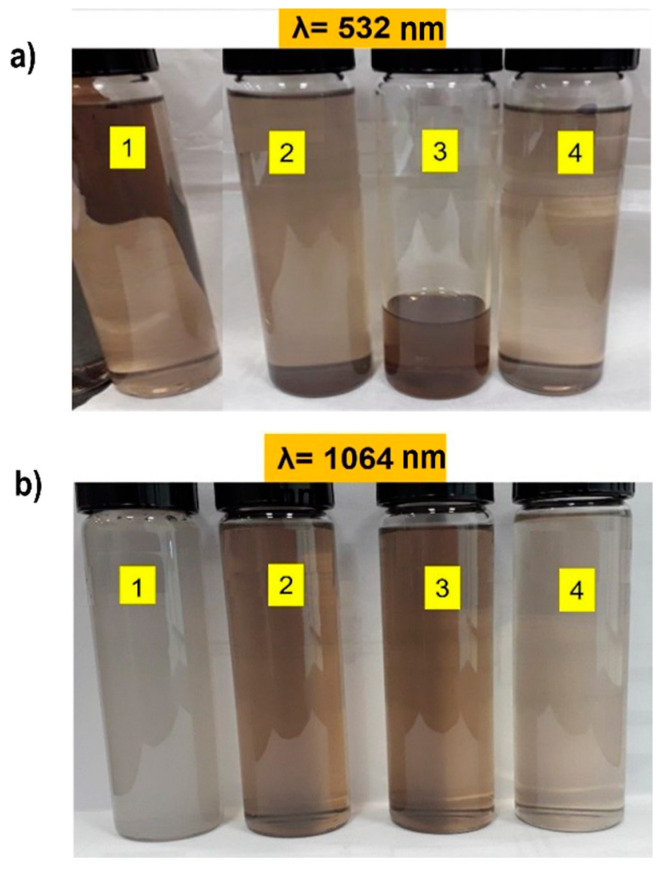
Photograph of BTO nanoparticles in different solvents at (**a**) λ = 532 nm and (**b**) λ = 1064 nm, where 1 = Distilled water, 2 = Isopropanol, 3 = Ethanol, and 4 = Acetone.

BTO nanoparticles (NPs) were obtained using the pulsed laser ablation in liquid media (PLAL) technique. For the synthesis process, a Nd:YAG LQ 929 pulsed laser, (Solar laser system, Minks, Bielorrusia), was used at its fundamental wavelength (1064 nm) and its first harmonic (532 nm), with a frequency of 10 Hz and 10 ns pulse width (Solar System, located in Facultad de Ingeniería Mecánica y Eléctrica, Apodaca, México). A target of bismuth titanium oxide (BTO) was used to produce BTO NPs in distilled water, ethanol, isopropanol, and acetone to evaluate the properties of the ablated products. The BTO target was immersed in 50 mL of each liquid in a glass beaker of 200 mL. The laser beam was focused perpendicularly to the target surface using a mirror (99% reflective) and a concave lens of 50 cm focal length. Laser fluence was calculated to be 25 and 12 mJ/cm^2^ for the laser operating at the fundamental wavelength and its second harmonic, respectively. A system of linear translation was employed to avoid local ablation and increase NP production and efficiency; the translation speed was set to 500 µm/s. The time of ablation was 1 min for every experiment. Figure 1 shows the photograph of BTO nanoparticles produced in different solvents at different ablation wavelengths of 532 nm (Figure 1a) and 1064 nm (Figure 1b). The color is indicative of the approximate size of the nanoparticles obtained; this is well reported, especially in metals [40,41,42]. Likewise, it is known that there is a notable relationship between size, shape, and optical properties, which prove to be very useful in a variety of high-performance applications.

In our work, the dark or tenuous nature of the solution is indicative of the concentration of the nanoparticles but not the size. Likewise, a brown color was observed in liquid media such as alcohols and in organic media; this could correspond to compounds that were formed (between organic media and NPs).

Morphology and crystal structure were analyzed using a TEM microscope, model FEI Titan G2 80-300 (Hillsboro, OR, USA, Centro de Investigación en Materiales Avanzados, Apodaca, México). To obtain TEM micrographs, a drop of each colloidal solution was placed in a TEM grid after ablation. To study crystal structure and elemental composition, precipitates of the obtained solutions were taken and placed in a silicon wafer and dried at room temperature until sufficient material for both analyses was gathered. X-ray diffractograms were obtained in a PANAlytical Empyrean diffractometer (Malvern, UK) using Cu Kα radiation (1.5405 Å), operating at 45 kV and 40 mA, in grazing incidence mode (Centro de Investigación en Materiales Avanzados, Apodaca, México). X-ray photoelectron spectroscopy (XPS, Thermo Scientific K-Alpha) was performed in a XPS Escalab 250Xi (Thermofisher, Waltham, MA, USA, Centro de Investigación en Materiales Avanzados, Apodaca, México) with monochromatized Al Kα radiation (E = 1486.68 eV). The recorded binding energy data were referenced to the binding energy of adventitious carbon (C 1s at 284.6 eV)). To analyze optical properties, the colloidal solutions were placed in a 1 cm path-length cuvette, and its reference in an UV-Vis absorption spectrophotometer (Shimadzu UV-1800, Tokyo, Japan, Centro de Investigación en Materiales Avanzados, Apodaca, México).

## 3. Results

### 3.1. Morphology

**Figure 2:**
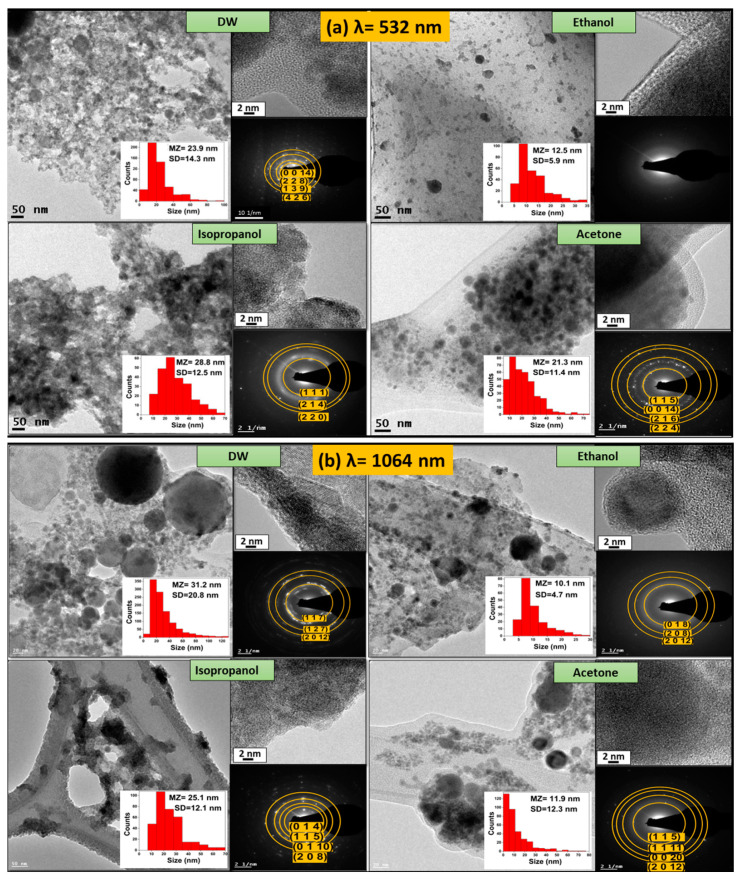
TEM micrographs corresponding to BTO nanoparticles synthesized by PLAL using (**a**) λ = 532 nm and (**b**) λ = 1064 nm.

Figure 2 shows TEM micrographs of the BTO nanoparticles obtained by PLAL in different liquid media with laser irradiation using (a) λ = 532 and (b) λ = 1064 nm, respectively. Figure 2 includes TEM images, high-resolution TEM (HRTEM), and selected area electron diffraction (SAED) for each liquid (distilled water (DW), ethanol, isopropanol, and acetone). Pulsed laser ablation of the BTO target produced spherical particles at both wavelengths, 532 and 1064 nm. At a laser wavelength of 532 nm (Figure 2a), nanoparticles produced by PLAL in distilled water, ethanol, isopropanol, and acetone showed spherical morphology with mean sizes (MZs) of 23.9, 12.5, 28.8, and 21.3 nm, respectively. At the fundamental wavelength of the Nd:YAG laser, 1064 nm (Figure 2b), PLAL synthesized spherical particles with a broader size distribution, with mean sizes of 31.2, 10.1, 25.1, and 11.9 nm, for NPs synthesized in distilled water, ethanol, isopropanol, and acetone. BTO nanoparticles at both wavelengths are clustered.

For the nanoparticles produced using λ = 532 nm, the SAED micrographs showed the presence of concentric rings, which corresponded to the following diffraction planes of orthorhombic bismuth titanium oxide (JCPDF No. 73-2181): NPs produced in DW showed the planes (0 0 14), (2 2 8), (1 3 9), and (4 2 6); NPs produced in isopropanol showed (1 1 1), (2 1 4), and (2 2 0); and NPs produced in acetone showed (1 1 5), (0 0 14), (2 1 6), and (2 2 4), all of them corresponding to the orthorhombic Bi_4_Ti_3_O_12_ (JCPDF No. 73-2181), while the nanoparticles produced in ethanol showed no planes, due to the amorphous nature of nanoparticles obtained by this medium. Moreover, BTO nanoparticles synthesized by PLAL using λ = 1064 nm in different media presented the following diffraction planes of orthorhombic BTO: in DW, (1 1 7), (1 2 7), and (2 0 12); in ethanol, (0 1 8), (2 0 8), and (2 0 12); in isopropanol, (0 1 4), (1 1 5), (0 1 10), and (2 0 8); and in acetone, (1 1 5), (1 1 11), (0 0 20), and (2 0 12).

Figure 2 shows the different sizes of nanoparticles in the different media used; however, with ethanol it is observed that the diameter of the nanoparticles produced is more uniform.

There is an excellent review on the variables that determine particle size, morphology, and properties of the resulting colloids via laser ablation in liquid media [43]. In short, at higher laser wavelengths, particle size is broader due to the low absorption of the material at the given wavelength (1064 nm). At shorter wavelengths, such as 532 nm, particle size distributions are narrower and the average particle size is smaller due to the higher absorption of the materials [44]. BTO absorbs light in the UV range (λ < 400 nm) [45], which explains this behavior. Also, the dipole moment from the solvent plays an important role in particle size distribution. Ethanol has a dipole moment of 1.69, which interacts with the electrostatic force from the electrical double layer from the nuclei and species of the plasma plume formed during the ablation process. Acetone has the highest dipole moment of 2.89. Therefore, this explains the different particle sizes and morphology changes of the obtained colloids in ethanol [46].

### 3.2. Crystal Structure

**Figure 3:**
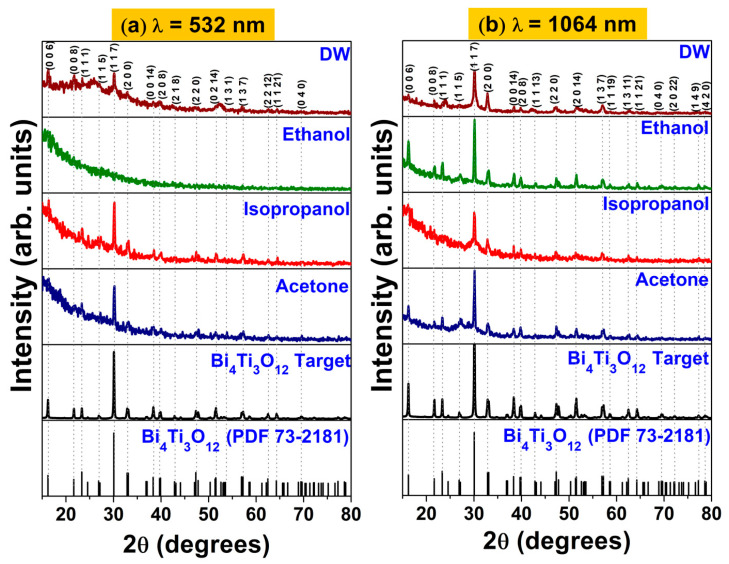
XRD corresponding to BTO nanoparticles synthesized by PLAL using (**a**) λ = 532 and (**b**) λ = 1064 nm.

Figure 3 shows the diffractogram of the BTO target and nanoparticles synthesized by PLAL using a wavelength of (a) λ = 532 nm and (b) λ = 1064 nm. The precursor target showed a polycrystalline nature, corresponding to the orthorhombic bismuth titanium oxide (JCPDF No. 73-2181). BTO nanoparticles synthesized by PLAL at λ = 532 nm (Figure 3a) for DW, isopropanol, and acetone preserved the polycrystalline nature. No signal was detected for the NPs produced in ethanol with 532 nm (see Figure 2a and Figure 3a with ethanol). There are reports on amorphous nanoparticles obtained via laser ablation in liquid media. Typically, oxygen affine transition metals produce amorphous nanoparticles, mostly in water. However, at higher wavelengths, there is a chance of photofragmentation due to the higher absorption of light of the already present products of ablation. Other processes such as interband excitation, multiphoton absorption, and photoionization can lead to break the thermodynamic equilibrium state, especially at shorter wavelengths. Also, the solvent properties play a role in the crystallinity of the nanoparticles; organic solvents yield amorphous nanoparticles when oxygen with a single bond is present due to the lower binding energy of the C-C bond compared to the C=O double bond [47].

Those produced in acetone and isopropanol have a preferred orientation in the (1 1 7) direction; see Figure 3a. Figure 3b depicts the diffractograms for the BTO NPs produced by PLAL at λ = 1064 nm produced in different media. The NPs synthesized in distilled water, ethanol, isopropanol, and acetone show a polycrystalline nature, with higher counts compared with those produced by PLAL at λ = 532 nm. Furthermore, BTO nanoparticles synthesized at λ = 1064 nm in every medium showed preferential orientation in the (1 1 7) direction.

### 3.3. Chemical State

**Figure 4:**
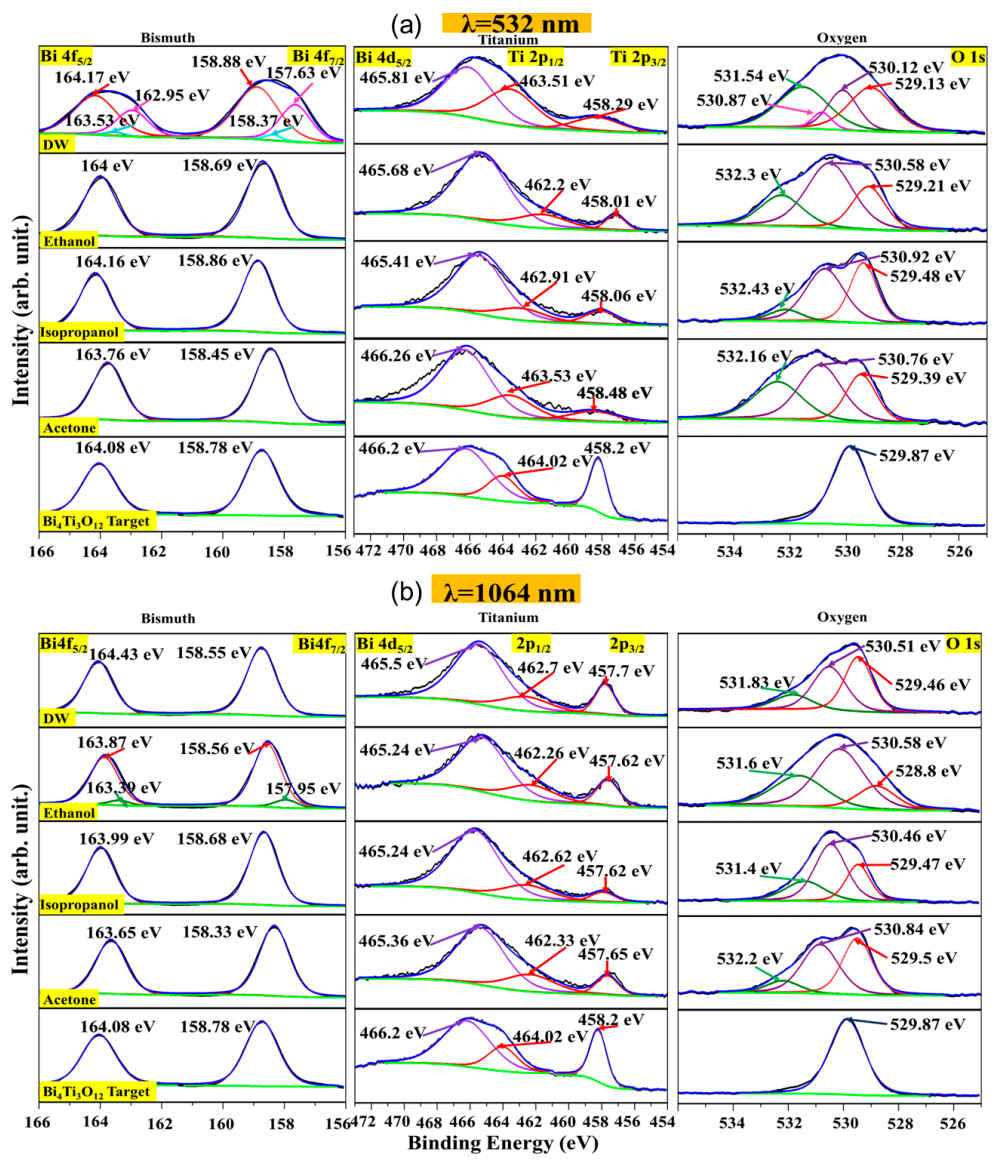
High-resolution XPS spectra of ablated nanoparticles produced by PLAL using different media using a laser wavelength with (**a**) λ = 532 nm and (**b**) λ = 1064 nm.

Figure 4a,b show the XPS core level spectra for λ = 532 nm and λ = 1064 nm of BTO nanoparticles in distilled water, ethanol, isopropanol, and acetone, respectively. To compare, the core levels of the target are shown as well. From Figure 4a,b, the binding energies corresponding to the elements in the lattice of the Bi_4_Ti_3_O_12_ target are: 158.78 eV for Bi 4f_7/2_, 458.2 for Ti 2p_3/2_, and 529.87 eV for O 1s, which are values in agreement with similar materials. It is important to note that a component for the Bi 4d_5/2_ core level appears on the Ti 2p spectra, which is expected for BTO, with a binding energy of 466.2 eV [19]. The peaks that are doublets possess spin-orbit components of 5.3 eV for Bi 4f, and 5.82 eV for Ti 2p [48]. After ablation, the binding energies for the peaks for Bi 4f decrease, which is also true for O 1s peaks. We present the discussion for the sample obtained with laser ablation for a 1064 nm laser in ethanol as the liquid media. In particular, the peak shape for O 1s is modified and new peaks appear at 530.58 eV and 531.6 eV, which are attributed to surface defects of oxygen [49]. It is important to note that the nanoparticles prepared on DW with the λ = 532 nm laser possess a component due to bismuth hydroxide at 158.88 eV and 531.54 eV for Bi 4f_7/2_ and O 1s, respectively. This is due to the solvent effects and laser energy. Some of the peaks of Bi 4f also possess a metallic component of 157.95 eV. These effects can be explained by a strong solvent effect and the high photon energy of the laser. Usually, properties such as refraction index, thermal properties, and flash point influence the final desired result of the nanoparticles [50]. It is important to note that the formation of bismuth oxides and hydroxides is very likely in a liquid medium such as distilled water. For example, Dasashi and co-workers determined the formation of different oxides and hydroxides of bismuth from a pure metallic target employing a λ = 1064 nm Nd:YAG laser. They found out that the nanoparticles interact strongly with the wavelength of the laser and the liquid media [51]. As such, it is expected that using a short-wavelength laser should induce the formation of bismuth hydroxides. The oxygen vacancies present after ablation are in agreement with other methods to induce oxygen vacancies, such as mechanical milling [52], chemical engineering [53], and sol-gel methods [54]. For the case of laser ablation in liquid media, the formation of oxygen vacancies can be obtained by varying the solvent used and the laser without employing long reaction times or producing harmful by-products. As such, the engineering of the vacancies can be facilitated by this method. It is important to note that the role of the vacancies play a fundamental part in applications such as magnetic materials [55]. Some Bi peaks also present a metallic component due to a reduction effect from the photon energy of the laser [56].

Table 1 and Table 2 show the XPS binding energies corresponding to the core levels for the nanoparticles obtained with λ = 532 nm and λ = 1064 nm lasers, respectively. The binding energies of the core levels corresponding to the target are also included.

### 3.4. Optical Properties

**Figure 5:**
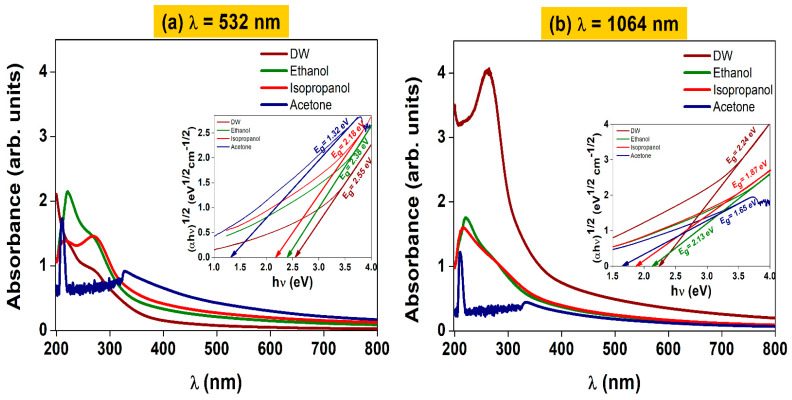
UV-Vis absorption of BTO nanoparticles synthesized via PLAL using laser wavelengths of (**a**) λ = 532 nm and (**b**) λ = 1064 nm in distilled water (DW), acetone, ethanol, and isopropanol.

UV-Vis spectroscopy was employed to study the optical absorption of the ablated BTO nanoparticles at both λ = 532 nm (Figure 5a) and λ = 1064 nm (Figure 5b). Figure 5a shows the absorption of BTO nanoparticles produced by laser irradiation at λ = 532 nm. The ablated nanoparticles show high absorption in the UV region of the electromagnetic spectrum. The liquid medium in which NPs are formed has an impact on the location of the absorption maximum. BTO nanoparticles obtained in distilled water have their maximum at 270 nm, while the nanoparticles in acetone present two maxima located at 211 and 328 nm. In a similar way, NPs produced in ethanol present absorption maxima at 221 and 271 nm, and nanoparticles produced in isopropanol have maxima at 218 and 267 nm. Figure 5b depicts the absorption of BTO nanoparticles formed by laser irradiation at λ = 1064 nm. BTO nanoparticles obtained in distilled water have their maximum at 270 nm, while the nanoparticles in acetone present two maxima, located at 211 and 328 nm. In a similar way, NPs produced in ethanol present absorption maxima at 221 and 271 nm, and nanoparticles produced in isopropanol have maxima at 218 and 267 nm. Figure 5b depicts the absorption of BTO nanoparticles formed by laser irradiation at λ = 1064 nm. These maxima are associated with the mean sizes of the clusters of NPs formed in the different media. Furthermore, the band gap (E_g_) of the BTO NPs was studied (inset inside of Figure 5a,b). Using a laser wavelength of 532 nm, the indirect band gaps were 2.55, 2.38, 2.18, and 1.32 eV for the BTO NPs produced by PLAL in DW, ethanol, isopropanol, and acetone, respectively. On the other hand, BTO NPs produced using a laser wavelength of 1064 nm showed indirect band gap energy of 2.24, 2.13, 1.87, and 1.65 eV for the NPs produced in DW, ethanol, isopropanol, and acetone, respectively. For the BTO NPs produced in DW, ethanol, and isopropanol at both laser wavelengths, the indirect optical bandgap transition can be demonstrated by photons in the visible range of the electromagnetic spectrum, while the nanoparticles produced in acetone present their indirect optical bandgap in the near-infrared region. The noise seen for both samples with acetone in Figure 5a,b from 220 nm to 330 nm is mainly due to two reasons:

1. This signal is characteristic of acetone (CH_3_CH_3_), since the energy radiation interacts with the bonds of acetone, absorbing energy at these wavelengths as reported in [57,58,59].
2. This noise also increases because acetone interacts with the material in the plasmonic region; having an electronic pair to share (that is, a carbon double bond of oxygen) confers high reactivity to the medium in this wavelength range donating electrons that react more with metals.

These results show that the BTO NPs produced by PLAL could have potential applications in photocatalysis, since their optical transition energy could be provided by the solar spectrum.

## 4. Conclusions

Bismuth titanate nanoparticles have been successfully synthesized via pulsed laser ablation in distilled water, ethanol, isopropanol, and acetone. The morphological, crystalline, and optical properties have been studied to evaluate the impact of the laser wavelength (532 nm and 1064 nm) and liquid medium on the properties of the BTO nanoparticles. TEM micrographs show that PLAL produced spherical particles, yet the sizes of the nanoparticles depended on the medium. SAED and XRD confirmed the polycrystalline nature of the BTO nanoparticles, with peaks associated with the orthorhombic phase of bismuth titanate. XPS spectra verified the chemical state of Bi, Ti, and O of each sample. Optical absorption spectra showed differences between the BTO NPs produced in different media and at different laser wavelengths. The optical bandgap values lie between 2.27 and 3.27 eV, depending on the medium and laser wavelength used.

## Figures and Tables

**Table 1:** XPS binding energies corresponding to the core levels for the nanoparticles obtained with a λ = 532 nm laser. The binding energies of the core levels corresponding to the target are also included.

Sample	Bi 4f	Ti 2p	O 1s
**DW**	4f_7/2_: 158.88 eV, 4f_5/2_: 164.17 eV 4f_7/2_: 158.37 eV, 4f_5/2_: 163.53 eV 4f_7/2_: 157.63 eV, 4f_5/2_: 162.95 eV	2p_3/2_: 458.29 eV, 2p_1/2_: 463.51 eV Bi 4d: 465.81 eV	531.54 eV 530.87 eV 530.12 eV 529.13 eV
**Ethanol**	4f_7/2_: 158.69 eV, 4f_5/2_: 164 eV	2p_3/2_: 458.01 eV, 2p_1/2_: 462.2 eV Bi 4d: 465.68 eV	532.3 eV 530.58 eV 529.21 eV
**Isopropanol**	4f_7/2_: 158.86 eV, 4f_5/2_: 164.16 eV	2p_3/2_: 458.06 eV, 2p_1/2_: 462.91 eV Bi 4d: 465.41 eV	532.43 eV 530.92 eV 529.48 eV
**Acetone**	4f_7/2_: 158.45 eV, 4f_5/2_: 163.76 eV	2p_3/2_: 458.48 eV, 2p_1/2_: 463.53 eV Bi 4d: 466.26 eV	532.16 eV 530.76 eV 529.39 eV
**Target**	4f_7/2_: 158.78 eV, 4f_5/2_: 164.08 eV	2p_3/2_: 458.2 eV, 2p_1/2_: 464.02 eV Bi 4d: 466.2 eV	529.87 eV

**Table 2:** XPS binding energies corresponding to the core levels for the nanoparticles obtained with a λ = 1064 nm laser. The binding energies of the core levels corresponding to the target are also included.

Sample	Bi 4f	Ti 2p	O 1s
**DW**	4f_7/2_: 158.55 eV, 4f_5/2_: 164.43 eV	2p_3/2_: 457.7 eV, 2p_1/2_: 462.7 eV Bi 4d: 465.5 eV	531.83 eV 530.51 eV 529.46 eV
**Ethanol**	4f_7/2_: 158.56 eV, 4f_5/2_: 163.87 eV 4f_7/2_: 157.95 eV, 4f_5/2_: 163.39 eV	2p_3/2_: 457.62 eV, 2p_1/2_: 462.26 eV Bi 4d: 465.24 eV	531.6 eV 530.58 eV 528.8 eV
**Isopropanol**	4f_7/2_: 158.68 eV, 4f_5/2_: 163.99 eV	2p_3/2_: 457.62 eV, 2p_1/2_: 462.62 eV Bi 4d: 465.24 eV	531.4 eV 530.46 eV 529.47 eV
**Acetone**	4f_7/2_: 158.33 eV, 4f_5/2_: 163.65 eV	2p_3/2_: 457.65 eV, 2p_1/2_: 462.33 eV Bi 4d: 465.36 eV	532.2 eV 530.84 eV 529.5 eV
**Target**	4f_7/2_: 158.78 eV, 4f_5/2_: 164.08 eV	2p_3/2_: 458.2 eV, 2p_1/2_: 464.02 eV Bi 4d: 466.2 eV	529.87 eV

## Data Availability

Not applicable.
